# Metabolic improvement in patients with acid sphingomyelinase deficiency following intravenous trehalose administration: an untargeted pharmacometabolomic study

**DOI:** 10.1186/s13023-025-04188-z

**Published:** 2026-01-07

**Authors:** Mahdieh Khoshakhlagh, Maede Hasanpour, Mehrdad Iranshahi, Javad Asili, Aida Tasbandi, Tannaz Jamialahmadi, Amirhossein Sahebkar, Milad Iranshahy

**Affiliations:** 1 Department of Medical Biochemistry, Faculty of Medicine, Mashhad University of Medical Sciences, Mashhad, Iran; 2 Biotechnology Research Center, Pharmaceutical Technology Institute, Mashhad University of Medical Sciences, Mashhad, Iran; 3 Department of Pharmacognosy, Faculty of Pharmacy, and Persian Medicine and Pharmacy Research Center, Tehran University of Medical Sciences, Tehran, Iran; 4 Department of Pharmacognosy, School of Pharmacy, Mashhad University of Medical Sciences, Mashhad, Iran; 5 Applied Biomedical Research Center, Basic Sciences Research Institute, Mashhad University of Medical Sciences, Mashhad, Iran; 6 Pharmaceutical Research Center, Pharmaceutical Technology Institute, Mashhad University of Medical Sciences, Mashhad, Iran; 7 Centre for Research Impact & Outcome, Chitkara College of Pharmacy, Chitkara University, Rajpura, Punjab India

**Keywords:** Acid sphingomyelinase deficiency, Intravenous trehalose, Metabolomic, GC-MS, Pharmacometabolomic study

## Abstract

**Background:**

Acid sphingomyelinase deficiency (ASMD) A and B, historically known as Niemann-Pick (NP) types A (NPA) and B (NPB), are life-threatening and rare inherited lysosomal storage disorders, caused by a deficiency in the acid sphingomyelinase enzyme activity. The negative outcome of this deficiency is the sphingomyelin (SM) accumulation in different organs and tissues. Trehalose is a natural disaccharide with neuroprotective and autophagy-inducing abilities that has recently been shown to improve clinical and biochemical features of patients with ASMD A/B. We previously showed that trehalose can reduce the serum levels of sphingomyelins and improve disease symptoms caused by lipid accumulation in ASMD A/B patients.

**Aim:**

The aim of this study was to investigate the serum metabolome changes in five patients with ASMD A/B, who received 15 g/week of trehalose intravenously for three months, using an untargeted gas chromatography-mass spectrometry (GC-MS) method.

**Methods and materials:**

GC-MS technique was used to assess the serum metabolic profile of patients with ASMD A/B. MSDIAL was used for data processing, and multivariate data analysis including Principal Component Analysis (PCA), and Orthogonal projections to latent structures discriminant analysis (OPLS-DA) algorithms were carried out using SIMCA.

**Results:**

OPLS-DA model revealed significant changes in several serum metabolites including phosphate (*P* = 0.0019), sorbitol (*P* = 0.00009), myoinositol (*P* = 0.02), threonine (*P* = 0.01), lactic acid (*P* = 0.0001), 1-monopalmitin (*P* = 0.01), threitol (*P* = 0.002), ribitol (*P* = 0.008), and D-ribose (*P* = 0.007) following trehalose treatment.

**Conclusion:**

The findings revealed that the beneficial effects of trehalose in patients with ASMD might be mediated by metabolic alterations. A clear shift in glucose metabolism in favor of less fatty acid production together with facilitating the breakdown of sphingomyelins is involved in the observed protective activity.

**Supplementary Information:**

The online version contains supplementary material available at 10.1186/s13023-025-04188-z.

## Introduction

Acid sphingomyelinase deficiency (ASMD) diseases are a class of lysosomal storage disorders, and impaired acid sphingomyelinase activity (ASM) underlies their clinical manifestations [1, 2]. Phosphocholine and ceramide are two main products of ASM resulting from the hydrolytic cleavage of sphingomyelin in lysosomes [3, 4]. Low enzyme activity characterizes the two main ASMD disease types: A and B. Patients with ASMD type A exhibit failure to gain weight and grow at the expected rate, hepatosplenomegaly, and progressive deterioration of the nervous system that leads to death before the first year of life [4–6]. Hepatosplenomegaly may be severe and lead to symptoms of liver failure in patients with ASMD type B, but they show no obvious symptoms of brain involvement [7–10]. The ASMD impairs the body’s ability to metabolize cholesterol and lipids within cells, leading to increased serum triglyceride and low-density lipoprotein (LDL) cholesterol levels and decreased high-density lipoprotein (HDL) cholesterol levels [11]. In some patients, eyes may also have a reddish-brown halo surrounding the macula, and rarely, a distinct cherry-red spot can be seen [12]. In ASMD types A and B, the liver, spleen, lymph nodes, adrenal cortex, lung airways, and bone marrow all contain large, fat-rich foam cells. Many patients with ASMD undergo bone marrow transplantation as a treatment option [13, 14]. Early diagnosis and treatment are integral to reducing disease outcomes and improving the quality of life in patients; however, bone marrow transplantation (BMT), enzyme replacement therapy (ERT), and other therapeutic approaches have shown limited impact on the overall survival of patients [13, 15–17].

Trehalose is a naturally occurring non-reducing disaccharide consisting of two glucose units bound by an α,α-1,1-glycosidic linkage [18]. It is widely found in various organisms such as bacteria, fungi, animals, and plants. In these organisms, trehalose provides cellular protection against stressors such as temperature changes, dehydration, and oxidative stress [19]. Trehalose has been approved by the Joint WHO/ FAO Expert Committee on Food Additives (JECFA) and the U.S. Food and Drug Administration (FDA) in 2000 and has been used as a safe food additive in Europe since 2001 [20]. Several preclinical studies and clinical trials [21, 22] have investigated the various biological activities, efficacy, and safety of trehalose [23–28]. Trehalose has been demonstrated to mitigate neurodegenerative diseases caused by lysosomal storage disorder and prevent neuronal damage [29, 30]. The neuroprotective effects of trehalose in both in vitro and in vivo experiments may be explained by its anti-aggregation, anti-inflammatory, and antioxidant capabilities as well as its ability to induce autophagy [31, 32]. Furthermore, trehalose prevents protein misfolding and promotes autophagy thereby inducing the clearance of accumulated and pathogenic proteins, such as mutant huntingtin, α-synuclein, and phosphorylated tau, in neurodegenerative diseases [31].

In our previous study, we investigated the impact of intravenous trehalose administration (15 g/week) for 12 weeks in patients with ASMD types A and B. Our findings demonstrated that trehalose was effective in reducing serum sphingomyelin levels and improving disease symptoms associated with lipid accumulation [33]. There has been rapid growth in the application of metabolomics approaches to identify the metabolites that play a role in disease pathogenesis and the therapeutic effects of various natural products across different diseases [34–38]. In this study, a gas chromatography-mass spectrometry (GC-MS)-based untargeted pharmacometabolomics approach was employed to explore the serum metabolic profile of patients in the referred trial was investigated before and after trehalose treatment.

## Materials and methods

### Reagents

The following reagents and standards were used: heptane (Sigma–Aldrich, Steinheim, Germany), 4–nitrobenzoic acid (Sigma–Aldrich, Steinheim, Germany), pyridine (Sigma–Aldrich, Steinheim, Germany), MilliQ^®^ water (Millipore, Billerica, MA, United States), O–methoxyamine HCl (Sigma–Aldrich, Steinheim, Germany) and MSTFA (N-Methyl-N-trimethylsilyl-trifluoroacetamide) with 1% TMCS (Trimethylchlorosilane) (Pierce Chemical Co., Rockford, IL, United States). Stearic acid methyl ester (C18:0 methyl ester) (Sigma–Aldrich, Steinheim, Germany) was used as an internal standard for GC–MS analysis, and the Kovats retention indices were calculated using alkane standard solution C8-C20 (Sigma–Aldrich, Steinheim, Germany).

### Study design and participants

This is a post-hoc metabolomics analysis of samples from a previously published clinical study [33]. The mentioned trial serves as the first proof-of-concept study evaluating the impact of intravenous trehalose administration in patients with lysosomal storage diseases. The selection of the trehalose dosage was based on the safety and efficacy demonstrated in previous phase I and II trials [39]. Previous phase I trials have demonstrated the safety and tolerability of trehalose doses up to 54 g [40]. The single-arm open-label study enrolled 5 male patients aged 2 to 12 years (mean age of 4.4 years), with diagnoses of ASMD types A and B conducted between June 2020 and March 2021. The disease was confirmed by a genetic diagnostic testing and clinical examinations. The sample size was determined based on practical considerations such as the number of available patients and the available resources.

The protocol of this trial was approved by the Ethics Committee of Mashhad University of Medical Sciences, registered in the Iranian Registry of Clinical Trials (Code: IRCT20130829014521N16). Before participating in the trial, the children’s parents or legal guardians signed an informed consent form. Pre-treatment baseline samples were collected from patients with ASMD, as each participant served as their own control. Then, the enrolled patients received an intravenous trehalose infusion (15 g) once a week for 90 min over 12 weeks of treatment. For this study, a 3-month duration was selected based on the duration previous studies with the same intravenous trehalose dose [41–44]. The 12-week duration was deemed sufficient to observe clinically relevant changes in disease-related metabolites and assess the initial efficacy of trehalose therapy.

Follow-up visits were also conducted weekly during the study period. After 12 weeks, blood samples from patients with ASMD treated with trehalose were collected, centrifuged and the serum was separated and aliquoted into 1.5 ml Eppendorf microtubes and stored at -80 ℃ until further experiments.

Serum alanine aminotransferase (ALT) and aspartate aminotransferase (AST) levels were quantified using a kinetic colorimetric assay to evaluate hepatic function at baseline and following trehalose treatment. Spleen and liver volumes were determined by ultrasonography and volumetric analysis at both time points. High-resolution computed tomography (HRCT) of the chest was performed to assess and compare pulmonary status between week 0 and week 12.

### Blood serum metabolite extraction

Serum samples (50 µl) were thawed at 4 ℃ for 30–60 min, and serum metabolites were deproteinized by adding 150 µL of 25 ppm 4-nitrobenzoic acid in cold solvent (water/ methanol, 1:9, *v*/*v*) in 1.5 ml Eppendorf tubes.

Each sample was mixed on a vortex mixer for 15 s, allowed to settle for 8 min, and then centrifuged at 13,000 rpm for 10 min at 4 ℃ to remove the precipitated protein. The supernatant was aspirated and dried under nitrogen. All dried samples were then mixed with methoxyamine HCl (50 µL, 20 mg/mL in pyridine), and the mixture was incubated for 1 h at 70 ℃. After this time, the derivatization of serum metabolites was continued by adding 50 µL of N-Methyl-N-(trimethylsilyl) trifluoroacetamide (MSTFA: TMCS 1%), vortexing for 30 s, and re-incubating for 1 h at 70 ℃. The samples were then centrifuged at 13,000 rpm for 8 min. The supernatant was transferred to a 450 µL internal microinsert tube, which was placed into a sample vial for GC-MS analysis, and then 100 µL of standard solution (10 ppm methyl stearate in heptane) was added to each sample.

### Quality control (QC) and blank samples preparation

For quality control (QC) sample preparation or a pooled QC sample, small aliquots of each serum sample (50 µl) were pooled into a labeled 2 ml microcentrifuge tube and thoroughly mixed. After that, QC samples were prepared for GC-MS analysis as described above. The process blank sample was prepared by the same method mentioned above for derivatization, with the only difference of using water instead of a serum sample, and the equipment blank sample was obtained by injecting of GC-MS-grade methanol before sample injection. All the peaks from the two types of blank samples were labeled as contamination and later removed from the samples dataset.

### GC–MS analysis

One microliter of each derivatized sample was injected into an Agilent 5,975 apparatus equipped with DB-5MS quartz capillary column (30 m * 0.25 mm* 0.25 μm, Agilent J&W Scientific, Folsom, USA) and PAL RTC 120 autosampler in splitless mode. To avoid systematic bias during the analysis, the sequence of sample injection was randomly arranged. Moreover, one QC sample was injected after every 5 samples. The injector temperature was set at 250 ℃ and the flow rate of helium (known as a carrier gas, 99.999%) was 1.0 mL/min. The oven temperature gradually increased from 80 ℃ to 340 ℃ at a constant rate of 10 ℃ /min. The electron ionization (EI) source was set at 70 eV. The temperature of the electron ionization source was 200 ℃. The solvent delay duration was 5 min, and the MS scanning range was 35–550 *m/z* (Agilent 5977 A series mass spectrometer).

### GC–MS data pre-processing

Data preprocessing is an important step in metabolomics investigations that affects the quality of the output data, the processing potential, and subsequently, the biological interpretation. Preprocessing of GC-MS raw data was performed using MS-DIAL software (Ver. 4.90) [45]. Initially, GC-MS chromatograms were exported to the “abf” format and imported into the program. Then, data pre-processing was performed in several steps, including peak picking, baseline correction, noise removal or reduction, smoothing, deconvolution, alignment, peak integration, normalization (using methyl stearate as an internal standard), and final output. Each step had several algorithms selected based on the type of data.

### Metabolite identification

Deconvoluted mass spectra were extracted from any resolved peak, and metabolites were identified using the NIST14 mass spectral library (NIST Version 2.0, NIST, USA). The retention time of alkanes with the number of carbon atoms from C8 to C20 was used to calculate retention indices (RI) and these alkanes were analyzed using the same GC-MS method. Metabolites were identified with an over 80% similarity threshold to main metabolites or derivatized metabolites, including trimethylsilylated and methoximated compounds, as well as RI match. Moreover, experimental identification was done based on retention time, spectral similarity, and comparison with HMDB.

### Data processing and statistical analysis

All variables with the Coefficient of Variation percent (CV%) of more than 20% based on the peak areas from QC samples were removed, and finally, a data set of the samples consisting of average retention time (RT) and peak areas of metabolites was imported into SIMCA-P software (version 14.1, Umetrics AB, Umeå, Sweden) for multivariate data analysis. The orthogonal projections to latent structures discriminant analysis (OPLS-DA) algorithm was used as the discriminant analysis model, and variables with variable influence on projection (VIP) values exceeding 1.5 in the OPLS-DA model were considered potential biomarkers or discriminant metabolites. Univariate statistical analysis (Paired T-test) was performed using GraphPad Prism 9, and metabolites with VIP > 1.5 and *P* < 0.05 (Paired t-test) were considered statistically significant. False Discovery Rate (FDR)-adjusted p-value were calculated using the Benjamini-hochberg method to control the false-positive rate [46]. Based on the data analysis result, the identified biomarkers were used to determine the metabolic pathways involved in disease and treatment, and network pathway analysis was therefore performed using MetaboAnalyst 5.0. A hypergeometric Test was selected as the enrichment method, and topology analysis was performed using Relative-betweenness Centrality.

## Results

### Clinical characteristics of patients

As has been described in our previous paper [33], five male patients with ASMD type A and B, aged 2 to 12 years, were enrolled. The mean age of patients was 4.4 ± 4.3 years, and the ratio of males to female was 3:2. TAPQOL score – a measure of patients’ health-related quality of life, was 42.6 ± 24.2 at baseline. Baseline liver and Spleen cranio-caudal diameters were 2.2 ± 1.1 and 142.8 ± 36.8, respectively. Baseline concentrations of serum lysosphingomyelin (lysoSM) and lysosphingomyelin-509 (lysoSM-509) were 30511.5 ± 6576.5 nmol/L and 72.4 ± 119.1 nmol/L, respectively. The mean age of patients was 4.4 years. As reported in our earlier publication, TAPQOL scores – a measure of patients’ health-related quality of life – increased in all patients at week 12 versus baseline. Trehalose administration was also associated with improvements in serum lyso-SM-509, lyso-SM level, hepatic transaminase, and the prooxidant-antioxidant balance levels [47]. All the patients received their doses according to the schedule and no patient dropped out of the study.

### GC-MS total ion chromatogram

The typical total-ion chromatograms of the serum samples analyzed by GC-MS in patients with ASMD before and after treatment with trehalose are shown in Figure S1. Based on the chromatogram, most metabolites are efficiently separated, and peaks are sharp with no tailing. In addition, no signs of bleeding, baseline drift or significant change in retention times were observed in the chromatograms (Figure S1).

### The metabolomic analysis of ASMD patients after treatment with trehalose

**Fig. 1:**
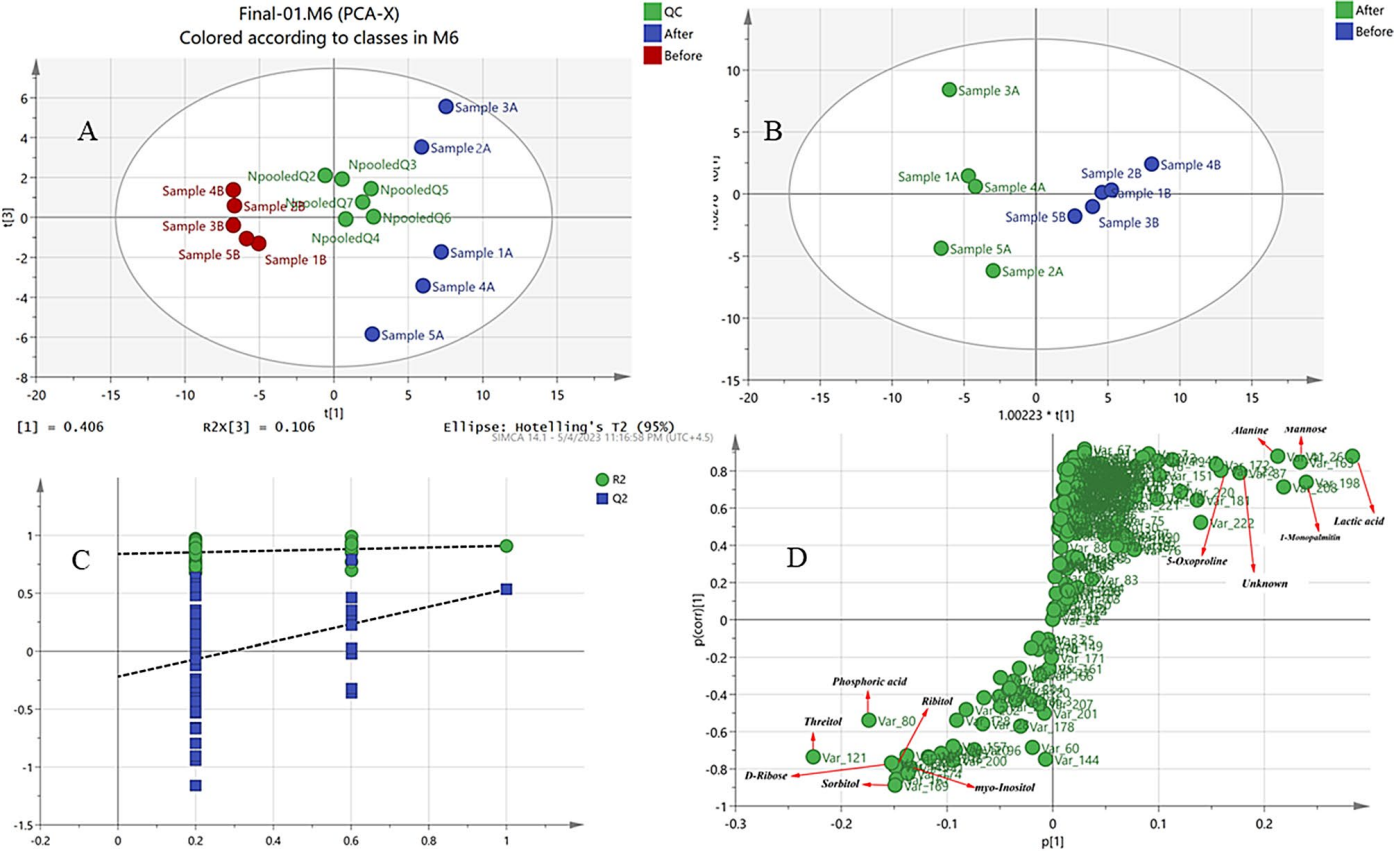
Multivariate data analysis of patients with acid sphingomyelinase deficiency before and after treatment with trehalose. (**A**) Principal component analysis (PCA) score plot of the patients before and after treatment with trehalose and pooled quality control (QC) samples. In the axis title, t means PC (**B**) Partial least squares discriminant analysis (PLS-DA) score plot of the patients before and after treatment with trehalose. (**C**) PLS-DA permutation test graph (permutation times *n* = 100) (**D**) S-plot after treatment with trehalose

The Principal Component Analysis (PCA) model as the most common multivariate data analysis is an unsupervised method to reduce the dimensionality of huge multivariate data sets [48]. As shown in Fig. 1A, the metabolic phenotypes of patients with ASMD before and after trehalose treatment showed distinct differences in the PCA model, indicating distinct serum metabolic fingerprints. Furthermore, the QC samples were densely distributed in the center of the score plots, indicating the reliability and sensitivity of the used method and the GC-MS.

The OPLS-DA model was used as a supervised method in the next step of multivariate statistical analysis to investigate the serum metabolic profiles of these groups and find potential metabolic markers. The OPLS-DA model provided better classification performance than the PCA model by filtering system noise and extracting variable information. The resulting score plot (Fig. 1B) of the OPLS‑DA model showed clear discrimination between serum metabolites of patients with ASMD before and after trehalose treatment. The resulting values of R2Y (cum) and Q2 (cum) of 0.909 and 0.533, respectively, indicate good fitness, discrimination, and predictive performance of the OPLS‑DA model. The loading plot (Figure S2) and S-plot (Fig. 1D) are useful for identifying the variable responsible for discrimination between groups. The assignment of these variables identified compounds responsible for separation in the score plot and considered biomarkers [49]. Moreover, to validate the OPLS-DA model, a permutation test with 100 permutations was performed, and the results showed that our model is valid for discriminating between two groups with a low risk of overfitting (Fig. 1C).

**Fig. 2:**
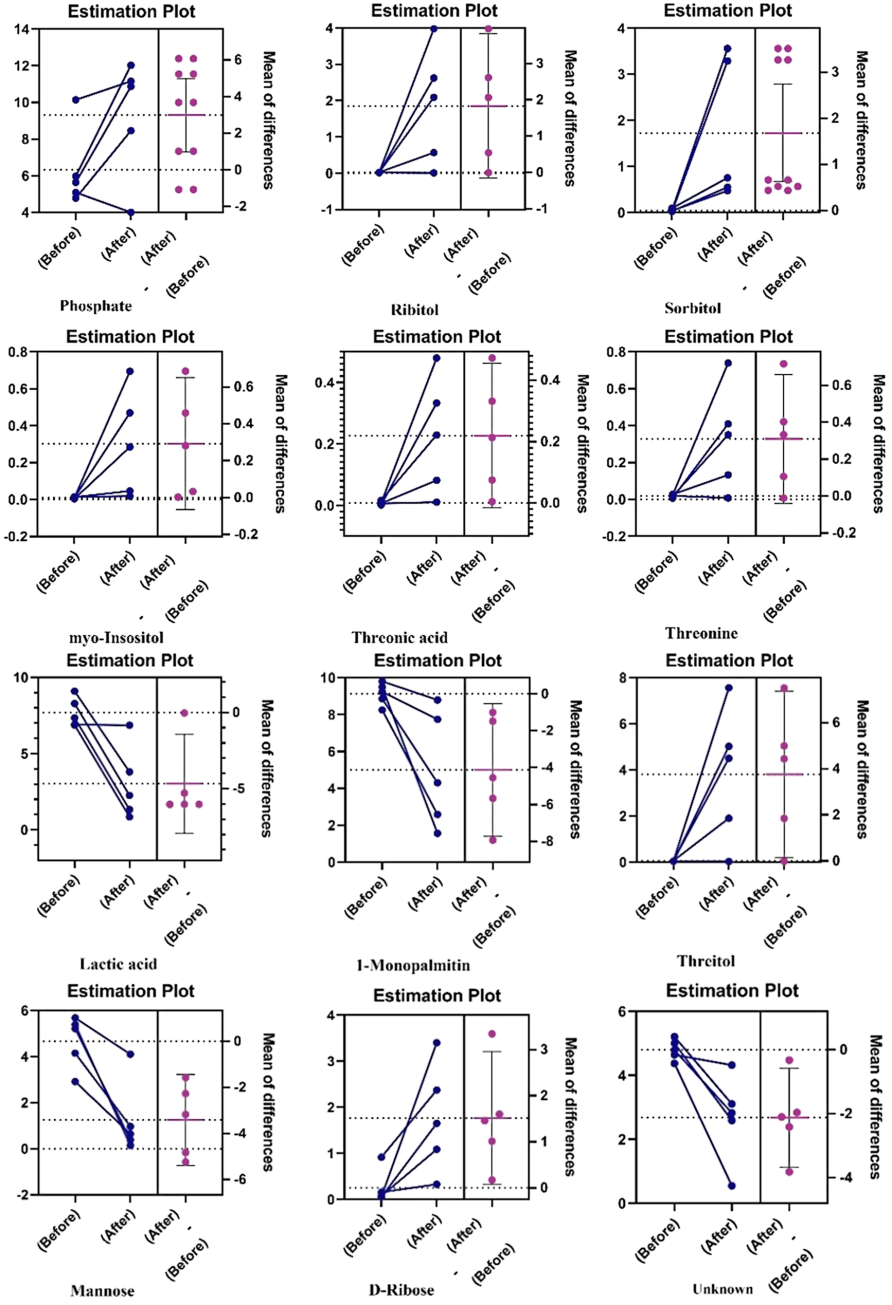
The estimation plots of biomarkers before and after treatment with trehalose using Paired T-test as univariate statistical analysis

Distinct metabolites were screened according to the VIP value of the OPLS-DA model, and variables with VIP value greater than 1.5 (Figure S4) were considered as key metabolites and biomarkers in the discriminant analysis of serum samples. Statistically significant metabolites were determined based on significant differences (VIP > 1.5 and FDR-adjusted *p* < 0.05), and only 12 metabolites with VIP greater than 1.5 had FDR-adjusted *p-value* lower than 0.05 (Table 1; Fig. 2).

**Table 1:** Major differential metabolites in the serum of patients with acid Sphingomyelinase deficiency before and after treatment with trehalose

Num.	RT (min)	Compound Name	Change	HMDB code	VIP	*p*-value	FDR adjusted *p*-value
1	7.42	Lactic acid	Decreased	HMDB0000190	3.749	0.0001	0.005
2	37.59	1-Monopalmitin	Decreased	HMDB0031074	3.474	0.01	0.046
3	15.04	Threitol	Increased	HMDB0004136	3.32	0.002	0.036
4	24.68	Mannose	Decreased	HMDB0000169	3.16	0.0006	0.011
5	10.71	Phosphate	Increased	HMDB0001429	2.89	0.001	0.027
6	11.44	Unknown	Decreased	-	2.4	0.003	0.036
7	19.98	Ribitol	Increased	HMDB0000508	2.12	0.008	0.045
8	15.82	Threonic acid	Increased	HMDB0000943	2.1	0.01	0.047
9	23.66	D-Ribose	Increased	HMDB0000283	2.05	0.007	0.045
10	25.15	Sorbitol	Increased	HMDB0000247	1.95	0.00009	0.006
11	27.19	myo-Inositol	Increased	HMDB0000211	1.87	0.02	0.048
12	13.00	Threonine	Increased	HMDB0000167	1.7	0.01	0.047

Abbreviations: RT, retention time; HMDB, Human Metabolome Data Base; VIP, Variable Importance in Projection; FDR: False Discovery Rate

The HMDB database was used to confirm the structure of metabolites associated with those variables. According to Fig. 2, lactic acid, Mannose, an unknown metabolite, and 1-monopalmitin significantly decreased in the serum of patients with Niemann–Pick disease after trehalose treatment. Ribitol, phosphoric acid, threitol, D-ribose, and sorbitol significantly increased in the serum of patients with ASMD disease after the treatment. These metabolites can be considered potential metabolic markers in the serum of patients with ASMD after trehalose treatment.

**Fig. 3:**
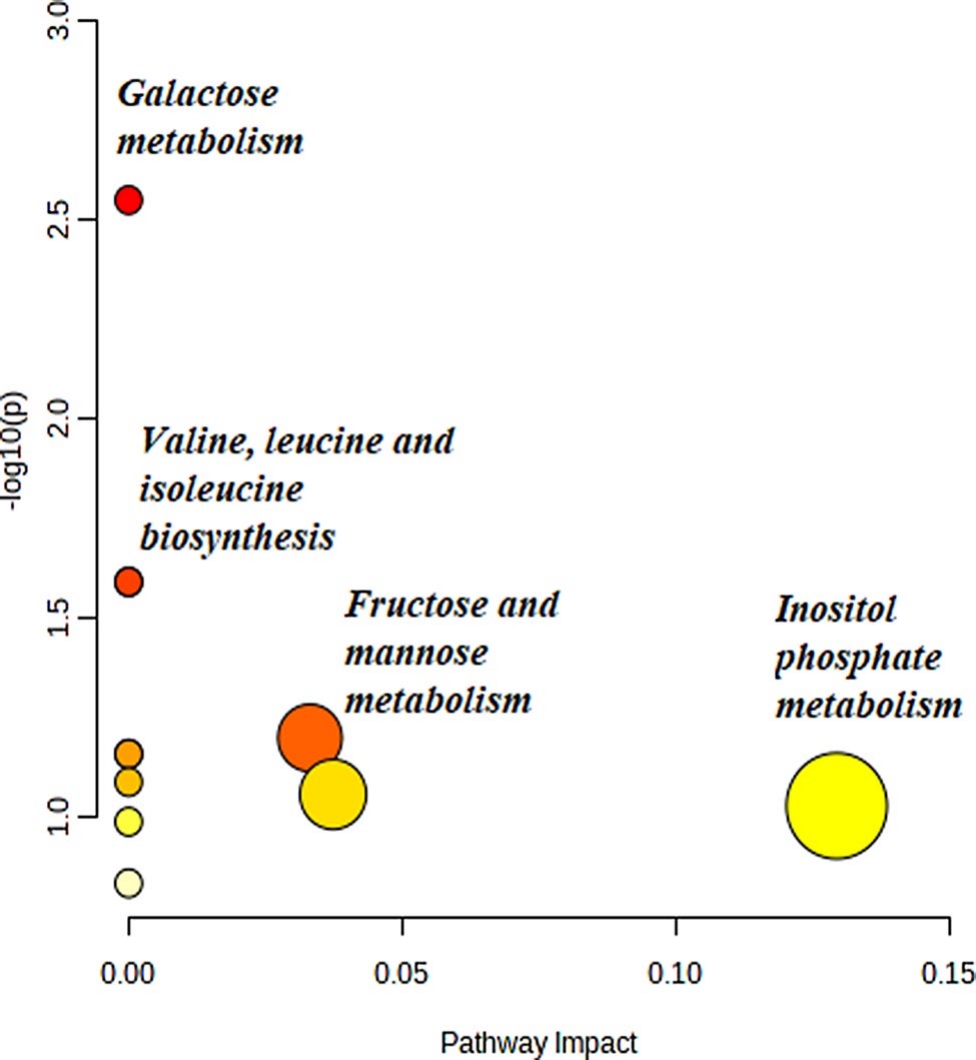
Metabolic pathway enrichment and topology analyses using the MetaboAnalyst platform, and 13 important features identified by t-tests (*p* < 0.05) comparing GC-MS data from acid sphingomyelinase deficiency patients before and after treatment with trehalose. Each data point of the graph represents a biologic pathway with quantified plasma metabolites

Metabolic pathway analysis revealed that most of these biomarkers might be related to galactose metabolism, valine, leucine, and isoleucine biosynthesis, fructose and mannose metabolism, and inositol phosphate metabolism (Fig. 3 and S3).

## Discussion

**Fig. 4:**
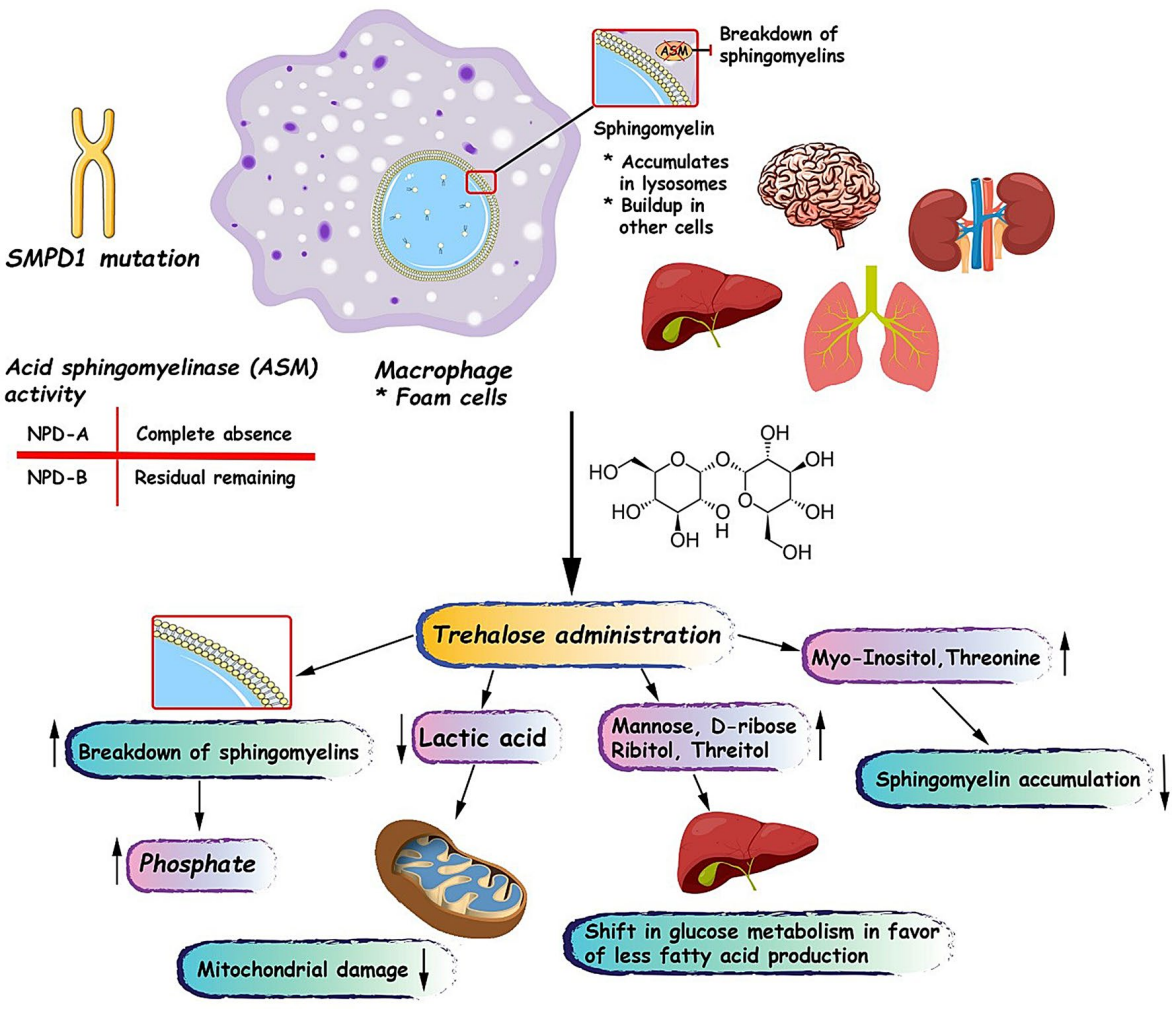
A schematic view of pathogenesis of acid sphingomyelinase deficiency and possible beneficial effects of trehalose administration in acid sphingomyelinase deficiency type A and B patients

Our findings suggest that trehalose administration in ASMD type A and B patients might be effective in the breaking down of sphingomyelins and improving overall cellular metabolism by protecting mitochondria. In addition, it seems that trehalose can alter glucose metabolism to favor reduced fatty acid synthesis and can also facilitate lipid breakdown (Fig. 4).

In this study, we observed a clear increase (about 2-fold) in the concentration of phosphate after treatment with trehalose. Increased plasma phosphate levels are mainly attributed to chronic kidney failure [50], whereas in our study, no adverse effects on kidney function were reported. We previously showed that trehalose administration can increase the breakdown of lyso-SM509 and lyso-SM [33]. Therefore, we hypothesize that the increase in phosphate level may be related to sphingomyelin degradation after the treatment. The breakdown of sphingomyelins typically occurs via hydrolysis of the phosphocholine headgroups, yielding ceramide and free phosphocholine [51]. In the next step, phosphocholine can be metabolized by the enzymes such as phosphoethanolamine/phosphocholine phosphatase to choline and phosphate [52]. Interestingly, in our previous study, we found that one of the patients (patient 5) did not benefit from the treatment at all in terms of lyso-SM509 and lyso-SM breakdown and even a slight increase in the level of these two sphingomyelins was observed [33]. Accordingly, in the current study, the patient’s phosphate level decreased after treatment, suggesting that the source of phosphate may be sphingomyelin degradation. In addition, only in three patients a marked decrease in sphingomyelin was observed, which is consistent with our findings for phosphate levels (Fig. 2).

Another metabolite that was significantly (*p*-value: 0.0001) altered following trehalose treatment was lactic acid. Lactic acid is a carboxylic acid that plays a crucial role in human cellular metabolism, particularly in glycolysis and the Cori cycle. It is produced by the enzymatic conversion of pyruvate by the enzyme lactate dehydrogenase (LDH). In aerobic conditions, pyruvate generated during glycolysis is further oxidized in the mitochondria via the tricarboxylic acid cycle and oxidative phosphorylation, yielding substantial ATP. However, under anaerobic conditions or during oxygen deprivation, such as intense exercise, hypoxia, or mitochondrial damage, pyruvate is converted into lactic acid to regenerate the NAD + required for glycolysis [53]. Mitochondrial dysfunction due to oxidative damage is a common pathological feature reported in several lysosomal storage diseases such as Gaucher and ASMD [54, 55]. We previously showed that trehalose administration has antioxidant activity and increases glutathione peroxidase (GPX) activity in the human body [33]. In this study, we observed a marked decrease in serum lactic acid level, which can be attributed to trehalose’s protective effect against mitochondrial damage. Moreover, facilitating sphingomyelin breakdown can partially reduce mitochondrial oxidative damage, as sphingomyelin deposition is linked to cellular stress responses, including endoplasmic reticulum stress, oxidative stress, and mitochondrial reactive oxygen species generation [56]. Interestingly, in one patient, lactic acid did not decrease, consistent with our previous findings that only 4 of 5 patients benefited from the treatment. Moreover, this pattern was observed for almost all other biomarkers. However, since lactic acid is not a direct biomarker of mitochondrial function, more evidence from measurements of mitochondrial biomarkers, such as mtDNA is needed to assess the true protective impact of trehalose on mitochondria.

Among the 12 assessed biomarkers, mannose and 1-monopalmitin showed significant decreases after the treatment. 1-Monopalamitin is a monoacylglycerol with an essential role in the production of ceramides in the human body, and ceramides are also the important building blocks of sphingolipids and sphingomyelins. Therefore, a reduction in 1-monopalmitin indicates that the treatment could shift overall metabolism towards less fatty acid production *via* the acetyl-CoA pathway [57–59]. These findings suggest that trehalose shifts glucose metabolism from glycolysis, which can produce lactic acid and acetyl-CoA, towards other pathways that use glucose as a source to produce other sugars such as mannose, D-ribose, ribitol, and threitol. This was evidenced by the increase in the serum levels of these four sugars.

A rise in myo-inositol serum concentration may explain another mechanism by which trehalose reduces sphingomyelin accumulation via myo-inositol’s lipotropic activity. Myo-inositol facilitates the removal of lipids and cholesterol from liver and myocardial cells [60].

Several changes in the serum level of amino acids were recorded, while only the change in threonine level was statistically significant. Animal studies have shown that threonine and lysine affect liver lipid composition, and the lower levels of these amino acids favor accumulation of lecithin, sphingomyelin, and free fatty acids [61]. Hence, an increase in threonine serum concentration may contribute to the sphingomyelin decrement observed in our study. However, more studies, and particularly clinical trials, are needed to establish the relationship between plasma threonine concentration and liver lipid composition.

Our study has several limitations, including a small sample size, a relatively short duration, the absence of a control group and the post-hoc design. Such limitations are inherent to every first-in-human and proof-of-concept study involving an intravenous intervention in the pediatric setting. The short treatment duration may affect the effectiveness and impact of treatment on serum metabolome. Although we checked several databases to identify biomarkers, achieving higher confidence requires chemical reference standards. Despite its high chromatographic resolution and reproducibility, GC–MS has several inherent limitations in metabolomics applications. Its metabolite coverage is restricted to volatile or derivatizable compounds, excluding many thermally labile or non-volatile metabolites such as large lipids and peptides. The derivatization process itself is often time-consuming and may introduce variability or chemical artifact.

## Conclusion

Overall, our findings revealed that trehalose induced clinical changes in several serum metabolites, including phosphate, sorbitol, and myoinositol. A clear shift in glucose metabolism toward reduced fatty acid production, together with facilitation of lipid and sphingomyelin breakdown, may be associated with the metabolic changes observed after trehalose treatment. This study justifies further metabolomics studies with more participants and longer durations in patients with ASMD A and B.

## Supplementary Information

Below is the link to the electronic supplementary material.

Supplementary Material 1

## Data Availability

The data that support the findings of this study are available from the corresponding author upon reasonable request.

## Abbreviations

ASMD: Acid sphingomyelinase deficiency
ASM: Acid Sphingomyelinase Activity
ATP: Adenosine Triphosphate
BMT: Bone Marrow Transplantation
CV: Coefficient of Variation
ERT: Enzyme Replacement Therapy
GC-MS: Gas Chromatography-Mass Spectrometry
HMDB: Human Metabolome Database
LDH: Lactate Dehydrogenase
MSTFA: N-Methyl-N-(trimethylsilyl) trifluoroacetamide
NAD+: Nicotinamide Adenine Dinucleotide
NPA: Niemann-Pick type A
NPB: Niemann-Pick type B
OPLS-DA: Orthogonal Partial Least Squares-Discriminant Analysis
PCA: Principal component analysis
QC: Quality Control
SM: Sphingomyelin
TMCS: Trimethylchlorosilane
VIP: Variable Interdependent Parameters
