# Supplementary material for: Metabolic improvement in patients with acid sphingomyelinase deficiency following intravenous trehalose administration: an untargeted pharmacometabolomic study
Source: Orphanet J Rare Dis. 2026 Jan 7;21:37. doi: 10.1186/s13023-025-04188-z (PMC12869916; doi:10.1186/s13023-025-04188-z)
Supplement: Supplementary file 1 — Supplementary Material 1 [file 13023_2025_4188_MOESM1_ESM.docx]

**Supplementary Information**

**Metabolic improvement in patients with Niemann-Pick disease following intravenous trehalose administration: An untargeted pharmacometabolomic study**

Mahdieh Khoshakhlagh^1ǂ^, Maede Hasanpour^2,3ǂ^ Mehrdad Iranshahi^2^, Javad Asili^4^, Aida Tasbandi^5^, Tannaz Jamialahmadi^6^, Amirhossein Sahebkar^2,5^, Milad Iranshahy*^4^

^1^ *Department of Medical Biochemistry, Faculty of Medicine, Mashhad University of Medical Sciences, Mashhad, Iran*

^2^ *Biotechnology Research Center, Pharmaceutical Technology Institute, Mashhad University of Medical Sciences, Mashhad, Iran*

*^3^Department of Pharmacognosy, Faculty of Pharmacy, and Persian Medicine and Pharmacy Research Center, Tehran University of Medical Sciences, Tehran, Iran.*

^4^ *Department of Pharmacognosy, School of Pharmacy, Mashhad University of Medical Sciences, Mashhad, Iran*

^5^ *Applied Biomedical Research Center, Mashhad University of Medical Sciences, Mashhad, Iran*

^6^ *International UNESCO center for Health-Related Basic Sciences and Human Nutrition, Mashhad University of Medical Sciences, Mashhad, Iran*

^ǂ^ These authors contributed equally to this work.

***Corresponding author:**

*Milad Iranshahy, Department of Pharmacognosy, School of Pharmacy, Mashhad University of Medical Sciences, Mashhad, Iran.*

E-mail: IranshahiML@mums.ac.ir


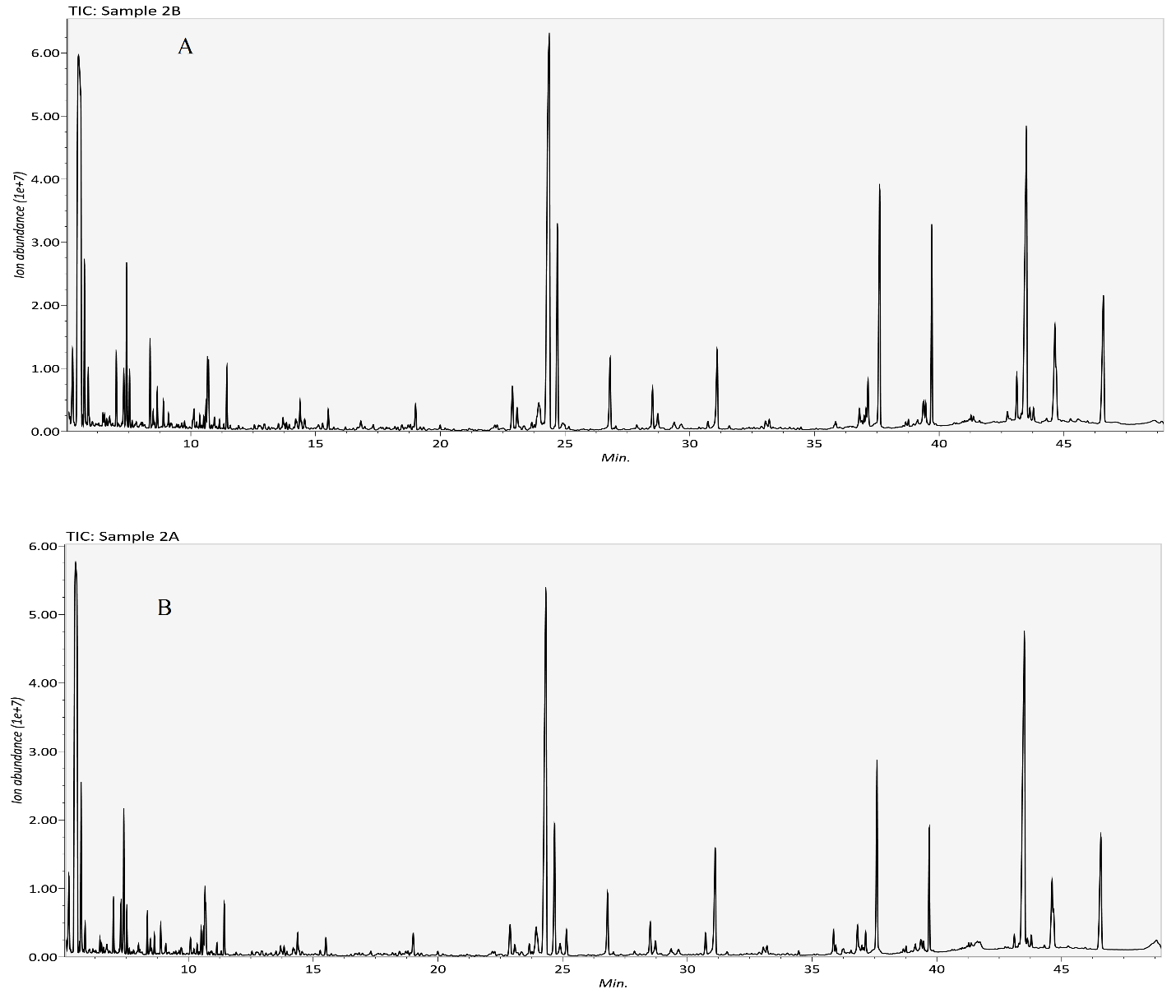


**Figure S1**. GC-MS TIC chromatogram of patients with Niemann-Pick disease before (A) and after (B) treatment with trehalose. Good separation and sharp peaks were recorded, and no sign of column bleeding has been observed. TIC, total ion chromatogram.


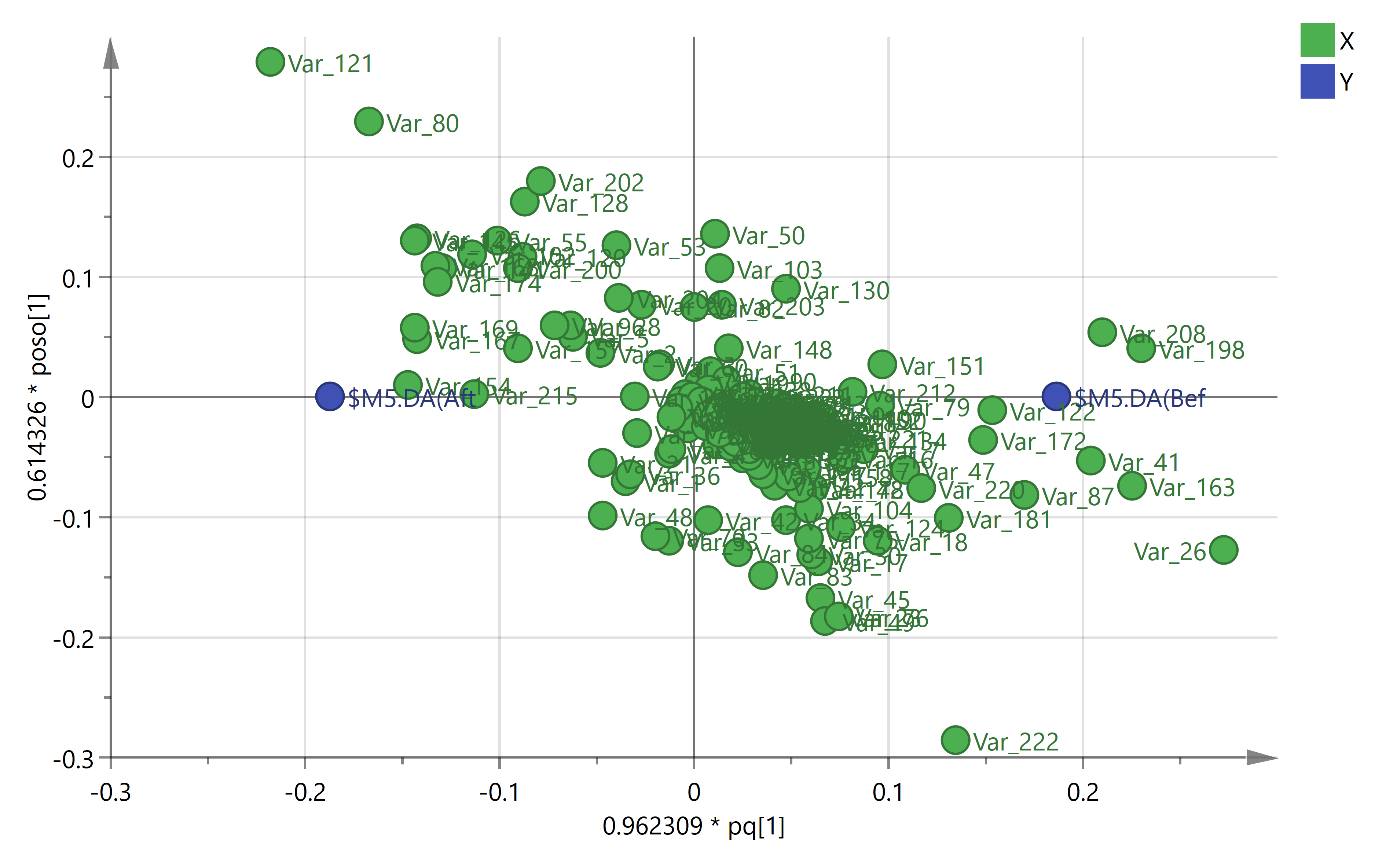


**Figure S2**. The OPLS-DA loading plot of Niemann-Pick patients serum samples before and after treatment with trehalose. The green circles are variables, and the blue circles are before (Bef) and after (Aft) treatment. OPLS‑DA, orthogonal projections to latent structures‑discriminate analysis.


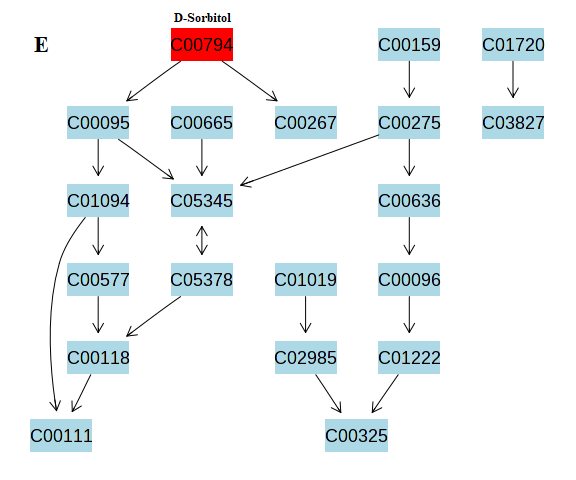

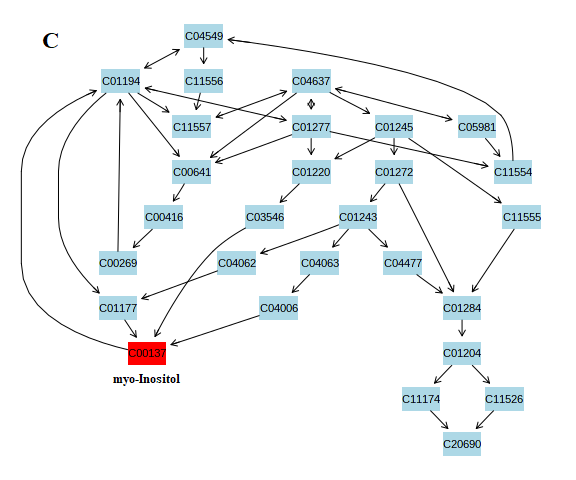

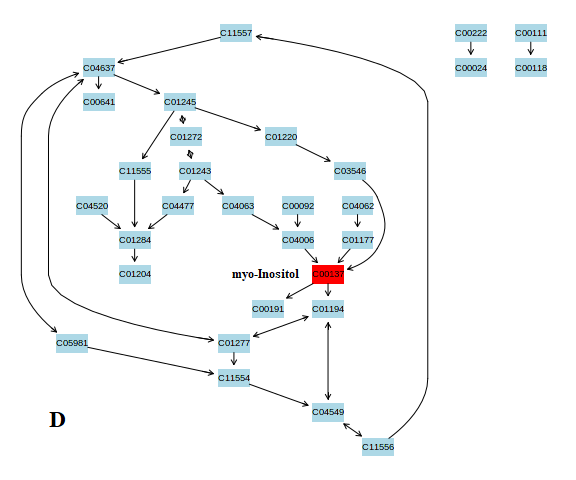

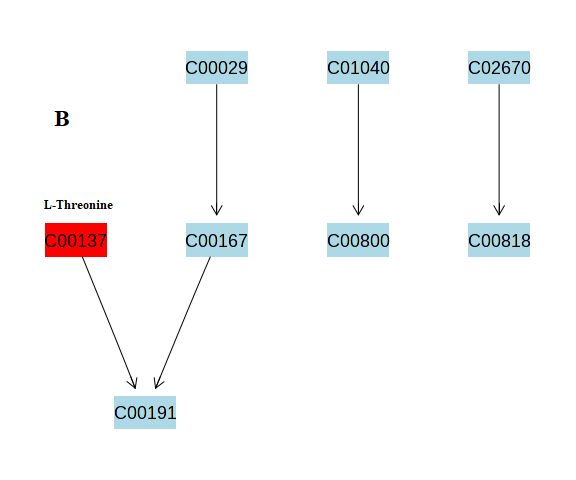

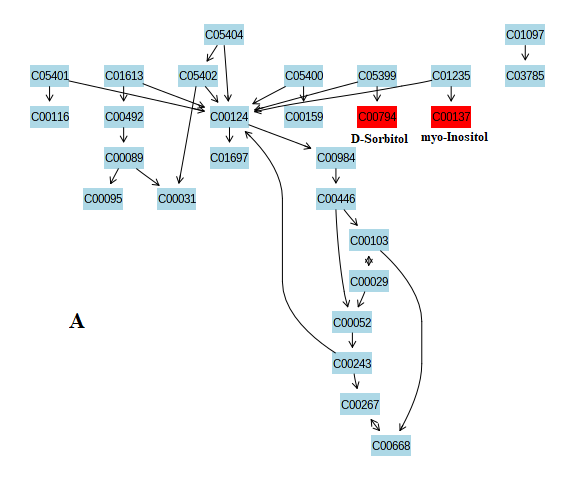


**Figure S3**. The results of pathway analysis for galactose metabolism (**A**), valine, leucine and isoleucine biosynthesis (**B**), phosphatidylinositol signaling system (**C**), inositol phosphate metabolism (**D**), and fructose and mannose metabolism (**E**) pathways.


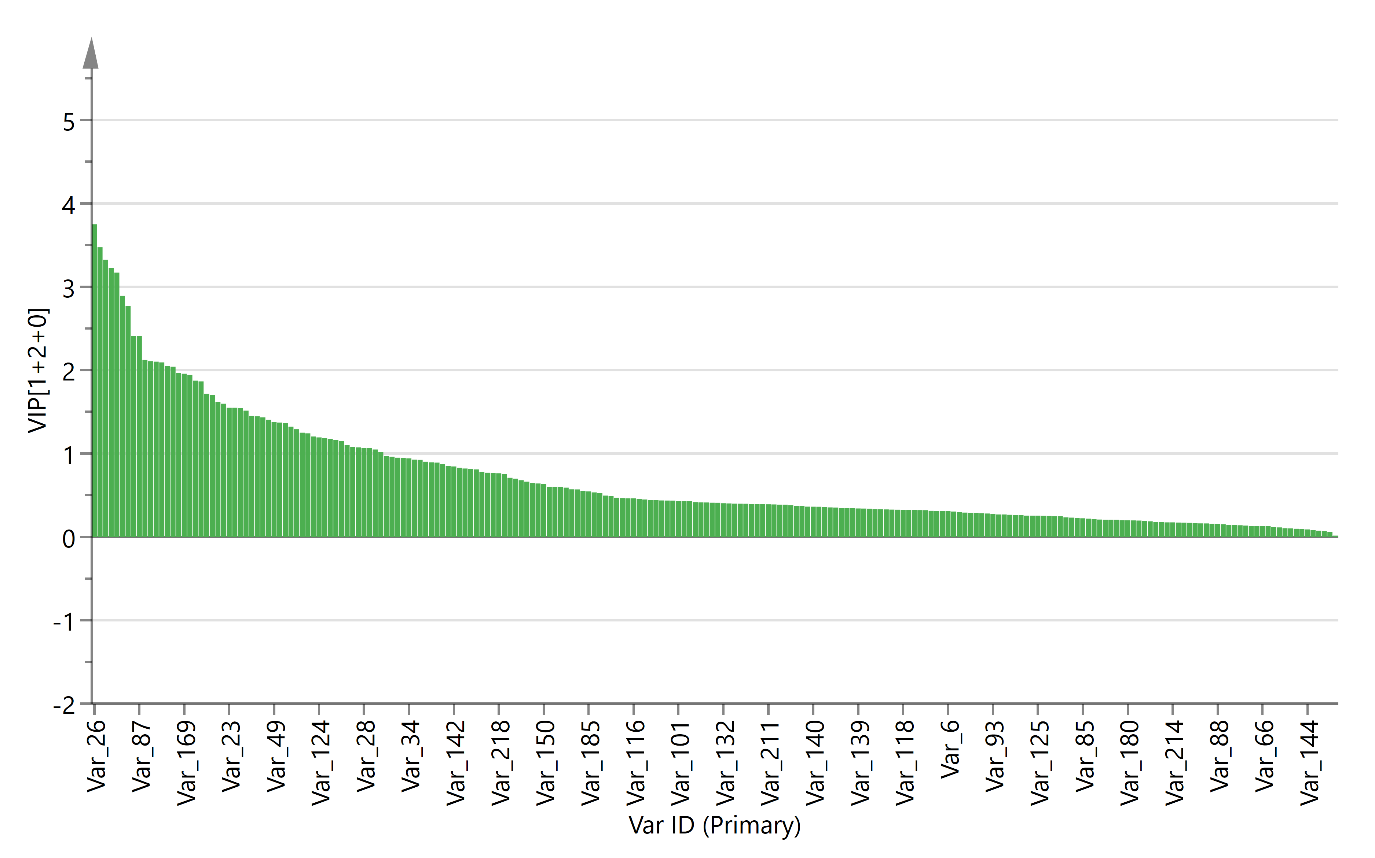


**Figure S4.** The OPLS-DA model Variable Importance for Projection (VIP) scores plot.
